# Can ITN distribution policies increase children’s ITN use? A DHS analysis

**DOI:** 10.1186/s12936-019-2824-9

**Published:** 2019-06-08

**Authors:** Katherine Theiss-Nyland, Jo Lines, Paul Fine

**Affiliations:** 1 Faculty of Infectious Disease Epidemiology, London School of Hygiene and Tropical Medicine, Keppel Street, London, WC1E 7HT UK; 2 Faculty of Infectious and Tropical Disease, London School of Hygiene and Tropical Medicine, Keppel Street, London, WC1E 7HT UK

**Keywords:** Malaria, Vector control, ITN, LLIN, Routine distribution, EPI, ANC

## Abstract

**Background:**

Insecticide-treated nets (ITN) have largely been distributed via mass distribution campaigns. Since 2011, however, the World Health Organization (WHO) has recommended additional ITN distribution via routine antenatal care (ANC) and expanded programme on immunization (EPI) services. Countries have begun to implement these routine facility-based distribution strategies, but inconsistently, and there is little research on outcomes of these new programmes. This paper investigates the impact of ITN distribution policies on children’s net use, comparing countries with different policies in place.

**Methods:**

Demographic Health Surveys from 25 countries in Africa were used to analyse household ITN ownership, and ITN use among children under 5 years of age. Countries were categorized in terms of the ITN facility-based distribution policies in place, based on nationally reported policies and distribution data provided to the WHO. The analysis was conducted for individual countries and then pooled with all countries in each category weighted equally to present the average country experience, by ITN distribution policy.

**Results:**

Household ITN ownership, children’s ITN use, and children’s ITN use in households with at least one ITN increase with each additional routine facility-based distribution policy. An average of 54.0% of children slept under an ITN in countries with ITN distribution via ANC and EPI, compared to 34.3% and 24.7% in countries with ITN distribution via ANC only, or no facility-based distribution, respectively. Linear regression found a 13% increase in net use among children under 5, on average, with each additional ITN distribution policy.

**Conclusion:**

ITN distribution via ANC and EPI can not only assist countries in maintaining ITN ownership and use, but may be extremely effective at increasing ITN ownership and use. There is also an additional benefit associated with combined ANC and EPI-based ITN distribution, compared to ANC distribution alone.

## Background

Long-lasting insecticidal nets (LLINs) are among the most effective tools for preventing malaria. As a result, LLINs have become the primary malaria prevention strategy recommended and championed by the World Health Organization (WHO) global malaria control programme [1]. Early mosquito net distribution efforts focused on pregnant women and children, because of their particular vulnerability to severe malaria morbidity and mortality. More recent strategies have focused on “universal coverage”, with the intention of providing ITNs for all people in areas with a high malaria burden. The WHO recommends household ownership of nets equal to one net for every 2 household members [2]. The Roll Back Malaria (RBM) strategic plan’s 2015 target stated that at least 80% of all members of populations at risk for malaria should be sleeping under an ITN on any given night [3, 4].

Since the mid-2000s, mass campaigns of free insecticide-treated nets (ITNs) have been the most common distribution strategy [5–7], accounting for 89% of the nets distributed in Africa [8]. The provision of free nets has dramatically increased ownership across Africa in the last decade.

Recognizing that campaigns are not enough to ensure high ITN coverage over time, new continuous distribution strategies have been developed to improve and maintain coverage in concert with campaigns. These strategies often focus on providing ITNs to the most vulnerable groups: pregnant women, infants and children. One such strategy, now recommended by the WHO, is the routine facility-based distribution of ITNs via both antenatal care (ANC) and childhood vaccination (EPI) programmes [9]. These recommendations specifically state that an ITN should be distributed to every woman at her first antenatal care visit in conjunction with other health interventions to assess and support the needs of a pregnant woman. By comparison, the recommendation for ITN distribution through EPI states that every child attending vaccination services should receive an ITN, but it does not specify at which vaccination visit within the first year of life the distribution should take place, leaving it to countries to make a decision for themselves. In most countries, there are at least five vaccination visits between birth and 1 year of age which could be used as a platform for ITN distribution.

Despite these recommendations, many countries have yet to implement routine facility-based ITN distribution, or have only implemented distribution via ANC. Distributing ITNs via ANC has been a significant topic of research, which finds that ANC distribution, in combination with campaigns, increases ITN ownership [10–12]. ANC has been the focus of routine facility-based distribution with the assumption that newborn babies will share a net with their mother, especially while they are breast feeding. ITNs distributed via ANC should, therefore, cover both the pregnant mother and the newborn child.

Some models developed to assess the benefit of different ITN distribution do not account for the increasing household size that results from a new birth [11]. While ITNs distributed via ANC may successfully replace campaign nets that have worn out between campaigns, new-born babies will increase the size of a household, thus increasing the total number of ITNs needed to cover all the household members. These growing households might benefit from additional ITN distribution through EPI. Research has yet to look at the benefits of ITN distribution through ANC and EPI together, as compared to ANC alone, a distribution strategy which could address some of these issues.

Analyses clearly show that ITN use is a function of ITN ownership: the more ITNs in a household, the more people sleep under an ITN [13]. There are also clear patterns by age across countries, with the highest use being by new-borns and pregnant women, and the lowest use by young adults 11–19 years of age [14]. The decline in net use with age begins in infancy. This analysis focuses on children under five, as one of the groups targeted by both ANC and EPI-based ITN distribution.

This research looks at the net use by children under-five comparing countries with no routine distribution through ANC or EPI, to countries with only ANC distribution, and countries with both ANC and EPI based distribution of ITNs.

## Methods

### Data

This analysis was conducted using data from the latest Demographic Health Surveys (DHS) in the quinquennium from 2010 to 2014 conducted in sub-Saharan Africa. Each DHS survey uses a multilevel cluster-sampling survey design in order to obtain a nationally representative sample of between 5000 and 30,000 households. The surveys consist of three questionnaires (household, women’s and men’s) and include interviews with all women in each household between the ages of 15 and 49 years, and a subset of men. The data are compiled into recoded datasets for use. Surveys were included in the analysis if information on type of mosquito nets present in the household and ITN use by household members were collected.

Children born to interviewed women within the 5 years preceding the survey, and alive at the time of the survey, were included in the analysis. The children’s recode and the household recode were used to include all variables of interest.

### Variable/definitions

#### ITN use

The main variable of interest for this analysis was the “use” of (i.e. sleeping under) a treated (either ITN or LLIN) bed net the night before the survey. Untreated nets were excluded.

#### Household access

Access is a measure of ITN availability within households, generally presented as the proportion of a population living in households with at least one ITN per two people per household. This variable was calculated using “de facto” household members in the household dataset: i.e. household members recorded as present the night preceding the survey. A household with at least one net per two household members, thus enough ITNs for all, is defined as having *universal access* [2].

#### Facility-based distribution policy

Countries were categorized as having no policy if they had not reported such a policy to WHO or had not reported any years of net distribution through their ANC or EPI programmes [8, 14]. Countries were categorized as having ANC and/or EPI-based distribution if they had reported such a policy to WHO and/or had more than one reported year of ITN distribution through these channels since 2010 [8, 14]. All countries with ANC and/or EPI-based distribution policies have had these policies since before 2010.

### Analysis

Household ITN ownership, and ITN use by children under-five years of age, is explored and stratified by the facility-based distribution policies present in countries.

For individual country analyses, internal weights from the DHS were maintained to provide nationally representative statistics, correcting for urban/rural and regional representation. For multi-country summaries and pooled analyses, all countries were weighted equally to present an average country statistic. Countries were weighted equally, rather than weighted by population to present the average experience countries might have under each policy decision. Analyses were presented either for all households, or for households with at least one ITN. All analyses were completed using Stata version 13 and 14. All the analyses accounted for the survey design, using either sampling weights or the svy commands in Stata.

## Results

Across all countries, the odds of ITN use generally decreased with age up to five (Table 1). A pooled analysis of all countries showed that children zero and 1-year-old had roughly the same level of ITN use, which decreased for 2, 3, and 4-year-olds respectively (Table 1). Gender in under-five year olds did not have any effect on ITN use, with the possible exception of Mali, Guinea and Rwanda. In Mali female children were less likely than their male counterparts to use ITNs: OR = 0.84 (0.74–0.95), while in Guinea and Rwanda female children were slightly more likely to use ITNS: OR = 1.24 (1.07–1.43) and OR = 1.11 (1.01–1.23) respectively (Table 1). However, recognising that this analysis involves multiple comparisons, there is an expectation that at least one will produce a significant difference by chance alone. A heterogeneity Chi squared for gender and ITN use across all countries was χ^2^ = 13.62, p = 0.96, supporting that conclusion and suggesting that there is no evidence of a significant difference between countries.

**Table 1:** Odds ratio for sleeping under a treated net for children aged 1–5 years compared to infants, and by gender, in houses with at least one ITN (unadjusted)

Country^a^	OR for net use given age					OR by gender	
	< 1 year	1 year old	2 years old	3 years old	4 years old	Male	Female
Benin	Ref	1.06 (.88–1.27)	1.00 (.83–1.20)	0.79 (0.66–0.93)	0.71 (0.59–0.84)	Ref	1.05 (0.94–1.18)
Burkina Faso	Ref	0.94 (0.79–1.11)	0.77 (0.65–0.99)	0.61 (0.52–0.70)	0.47 (0.40–0.55)	Ref	1.06 (0.96–1.17)
Burundi	Ref	1.30 (1.00–1.69)	0.67 (0.53–0.84)	0.58 (0.47–0.72)	0.46 (0.36–0.59)	Ref	1.00 (0.84–1.16)
Cameroon	Ref	0.96 (0.72–1.27)	0.81 (0.63–1.04)	0.50 (0.39–0.65)	0.54 (0.41–0.71)	Ref	0.92 (0.77–1.11)
Congo	Ref	1.12 (0.75–1.68)	0.86 (0.66–1.13)	1.02 (0.74–1.42)	0.87 (0.57–1.33)	Ref	0.82 (0.64–1.04)
Cote d’Ivoire	Ref	1.14 (0.94–1.38)	0.94 (0.78–1.13)	0.78 (0.63–0.97)	0.74 (0.61–0.91)	Ref	0.98 (0.85–1.13)
DRC	Ref	0.99 (0.82–1.19)	0.73 (0.61–0.87)	0.56 (0.47–0.67)	0.51 (0.43–0.60)	Ref	0.91 (0.82–1.01)
Gabon	Ref	0.64 (0.42–0.98)	0.59 (0.39–0.90)	0.53 (0.35–0.79)	0.43 (0.28–0.66)	Ref	1.13 (0.86–1.49)
Ghana	Ref	0.76 (0.61–0.95)	0.75 (0.59–0.96)	0.70 (0.55–0.90)	0.58 (0.46–0.73)	Ref	0.86 (0.74–1.00)
Guinea	Ref	1.08 (0.86–1.37)	0.88 (0.71–1.10)	0.90 (0.70–1.14)	0.65 (0.53–0.80)	Ref	1.24 (1.07–1.43)
Kenya	Ref	0.93 (0.79–1.10)	0.76 (0.64–0.90)	0.56 (0.47–0.65)	0.57 (0.48–0.67)	Ref	0.86 (0.78–0.95)
Liberia	ref	0.65 (0.50–0.84)	0.55 (0.43–0.70)	0.50 (0.37–0.68)	0.43 (0.34–0.54)	Ref	1.05 (0.89–1.25)
Malawi	Ref	1.00 (0.87–1.15)	0.82 (0.72–0.94)	0.78 (0.67–0.90)	0.66 (0.57–0.77)	Ref	1.10 (1.00–1.21)
Mali	Ref	1.07 (0.87–1.30)	0.82 (0.67–1.00)	0.67 (0.56–0.80)	0.57 (0.48–0.69)	Ref	0.84 (0.74–0.95)
Mozambique	Ref	0.93 (0.77–1.14)	0.76 (0.63–0.93)	0.62 (0.52–0.73)	0.53 (0.44–0.65)	Ref	0.99 (0.88–1.12)
Namibia	Ref	0.76 (0.50–1.16)	0.74 (0.1–0.96)	0.63 (0.41–0.96)	0.60 (0.37–0.98)	Ref	0.83 (0.61–1.13)
Nigeria	Ref	0.93 (0.82–1.06)	0.90 (0.81–1.00)	0.75 (0.67–0.83)	0.65 (0.57–0.74)	Ref	1.06 (0.98–1.15)
Rwanda	Ref	1.13 (0.94–1.35)	0.89 (0.75–1.06)	0.65 (0.55–0.77)	0.58 (0.49–0.69)	Ref	1.11 (1.01–1.23)
Senegal	Ref	1.07 (0.92–1.25)	1.01 (0.87–1.16)	0.93 (0.82–1.06)	0.91 (0.78–1.07)	Ref	1.06 (0.96–1.18)
Sierra Leone	Ref	1.05 (0.86–1.31)	1.03 (0.83–1.29)	0.87 (0.71–0.83)	0.66 (0.53–0.83)	Ref	1.02 (0.90–1.16)
Tanzania	Ref	1.10 (0.88–1.36)	1.00 (0.81–1.23)	0.91 (0.75–1.11)	0.96 (0.80–1.15)	Ref	1.07 (0.93–1.23)
Togo	Ref	0.89 (0.73–1.09)	0.79 (0.64–0.97)	0.71 (0.59–0.87)	0.79 (0.64–0.99)	Ref	1.03 (0.91–1.16)
Uganda	Ref	1.13 (0.91–1.40)	0.78 (0.64–0.95)	0.74 (0.60–0.90)	0.73 (0.58–0.92)	Ref	1.02 (0.89–1.16)
Zambia	Ref	0.93 (0.79–1.10)	0.75 (0.65–0.87)	0.59 (0.50–0.70)	0.53 (0.45–0.63)	Ref	0.97 (0.87–1.08)
Zimbabwe	Ref	1.05 (0.74–1.50)	0.94 (0.68–1.30)	0.83 (0.57–1.19)	0.72 (0.51–1.01)	Ref	1.16 (0.95–1.41)
All countries pooled	Ref	0.99 (0.95–1.04)	0.86 (0.82–0.90)	0.76 (0.73–0.80)	0.68 (0.65–0.71)	Ref	1.00 (0.97–1.03)

^a^Countries presented alphabetically

### Routine facility-based distribution policy

The proportion of total households owning at least one ITN was greater in countries where there was routine distribution of ITNs through both ANC and EPI (average = 64.45%), as compared to ANC alone (average = 55.10%) or neither (average = 39.28%), (Table 2). Furthermore, in countries with both ANC and EPI based distribution of ITNs, an average of 28.12% of households achieved universal access: enough nets for all household members, calculated as one net per two individuals (Table 2). By comparison, only 21.93% and 17.03% of households reached universal access in countries with ANC-based distribution, or no facility-based distribution, respectively (Table 2).

**Table 2:** Household ITN ownership by ITN distribution policy

Facility-based distribution policy	Country	DHS year	# of households surveyed	Living children under 5 years	% households with at least 1 ITN	% households with universal access^a^
ANC and EPI	Benin	2011	17,422	12, 679	80% (78.7–80.8)	45% (43.4–45.8)
	Burkina Faso	2010	14,424	13,716	57% (55.3–58.6)	19% (17.4–19.6)
	Burundi	2010	8596	7231	52% (48.8–55.2)	24% (21.2–25.9)
	Cote d’Ivoire	2012	9686	7093	67% (64.6–69.8)	32% (29.9–33.5)
	DRC	2012	18,171	17,228	70% (67.6–72.2)	25% (23.8–26.9)
	Gabon	2012	9755	5747	36% (34.1–38.2)	15% (13.4–15.6)
	Kenya	2014	36,430	20,093	59% (57.7–60.2)	35% (33.4–35.6)
	Malawi	2010	24,825	18,360	57% (55.5–58.1)	20% (19.0–20.9)
	Mali	2012	10,105	9582	84% (83.1–85.6)	42% (40.2–43.5)
	Rwanda	2010	12,540	8484	82% (80.1–83.2)	40% (38.7–42.0)
	Sierra Leone	2013	12,629	10,618	65% (62.3–66.5)	15% (14.0–16.0)
	Tanzania	2010	10,300	7526	64% (62.1–65.5)	23% (21.8–24.3)
	Togo	2013	9549	6535	65% (63.6–67.1)	33% (31.3–34.6)
Average					64.5%	28.1%
ANC only	Guinea	2012	7109	6424	47% (45.2–49.6)	10% (8.8–10.8)
	Liberia	2013	9333	7058	55% (51.6–57.6)	22% (20.4–23.9)
	Mozambique	2011	13,919	10,291	52% (49.5–53.3)	23% (21.1–24.1)
	Nigeria	2013	38,522	28,596	50% (47.7–51.4)	22% (21.0–23.3)
	Uganda	2011	9033	7355	60% (57.7–61.9)	28% (26.1–29.4)
	Zambia	2013	15,920	12,714	68% (66.3–69.1)	27% (26.2–28.5)
Average					55.1%	21.9%
No continuous distribution	Cameroon	2011	14,214	10,734	18% (17.4–19.2)	5% (4.1–5.0)
	Congo	2011	11,632	8857	33% (31.2–35.0)	11% (10.0–12.2)
	Ghana	2014	11,835	5595	68% (66.7–70.0)	45% (43.6–46.9)
	Namibia	2013	9849	4818	24% (23.0–25.9)	12% (11.0–13.1)
	Senegal	2010	7902	11,633	63% (59.8–66.0)	17% (15.7–18.5)
	Zimbabwe	2010	10,828	5203	29% (26.1–31.7)	12% (10.7–14.2)
Average					39.3%	17.0%

^a^Proportion of HH with enough nets for all HH members, defined as one net per two people per household

**Fig. 1:**
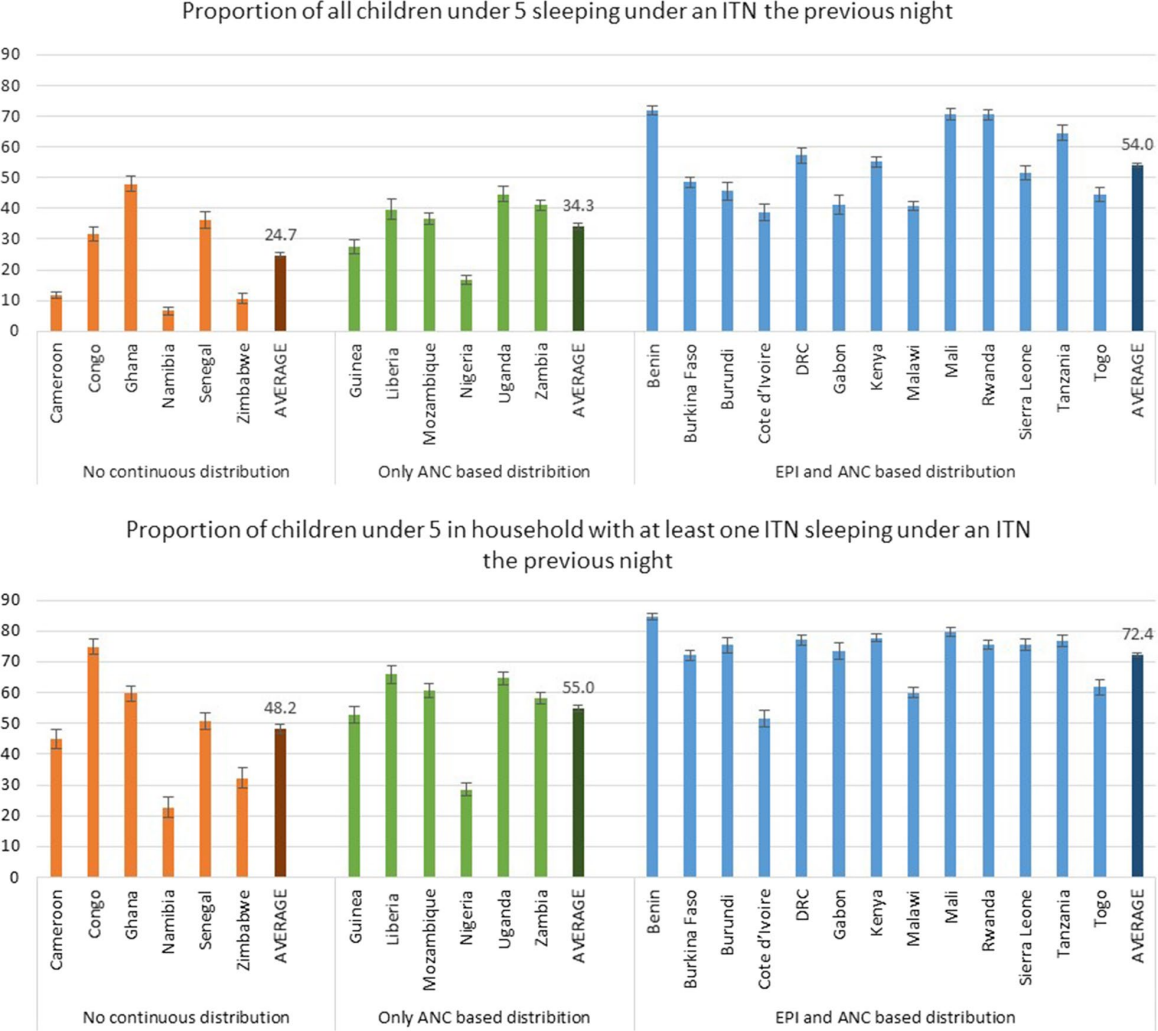
ITN use by children under 5 years, by distribution policy. Simple average used with all countries weighted equally to create an “average country” not “average individual”

**Fig. 2:**
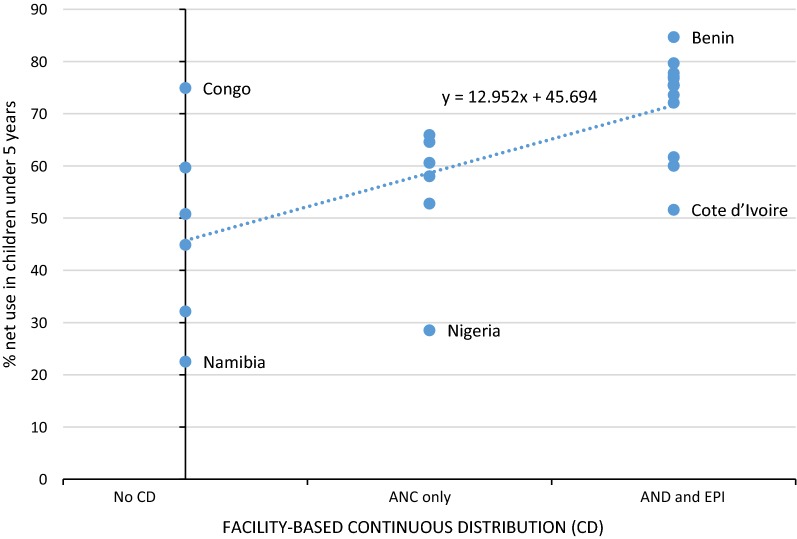
Best fit linear regression of ITN use in children under 5, stratified by facility-based distribution policies. Each dot represents one country proportion of ITN use in children under five, in households with at least one ITN. The best fit line produces an equation of y = 12.95x + 45.69 where the increase in net use with each additional policy equals 12.95% (6.81–19.09) and 45.69 (36.34–55.05) represents the estimated proportion of children using an ITN in the category of no continuous distribution policy. Outlier countries have been labelled for convenience

Children under-five in countries with both ANC and EPI-based ITN distribution were more likely to be sleeping under an ITN than children in countries with only ANC based distribution or with neither (Fig. 1). The average proportion of children sleeping under an ITN in countries with both distribution channels was 54.0% (53.3–54.6), compared to 34.3% (33.3–35.2) in countries with only ANC based distribution, and 24.7% (23.9–25.6) in countries with neither distribution channel (Fig. 1). Excluding households with no ITNs, on average 72.4% (71.8–73.0) of children slept under ITNs in countries with both ANC and EPI based distribution, compared to averages of 55.0% (53.9–56.0) and 48.2% (46.8–49.5) in countries with ANC-only and no facility-based distribution, respectively (Fig. 1). A best-fit line for children’s ITN use in households with at least one net predicts a 13.0% (6.8–19.1) increase in net use with the addition of each facility-based distribution policy (Fig. 2).

Using logistic regression and controlling for maternal education and household size in all countries, the likelihood of a child sleeping under an ITN increases almost twofold (OR = 1.92; 95% CI 1.86–1.97) for each additional distribution policy added. If the analysis is restricted to households with at least one ITN, children are OR = 1.68 (95% CI 1.63–1.73) times as likely to use an ITN with each additional policy, as compared to the children living in countries with one less policy.

**Fig. 3:**
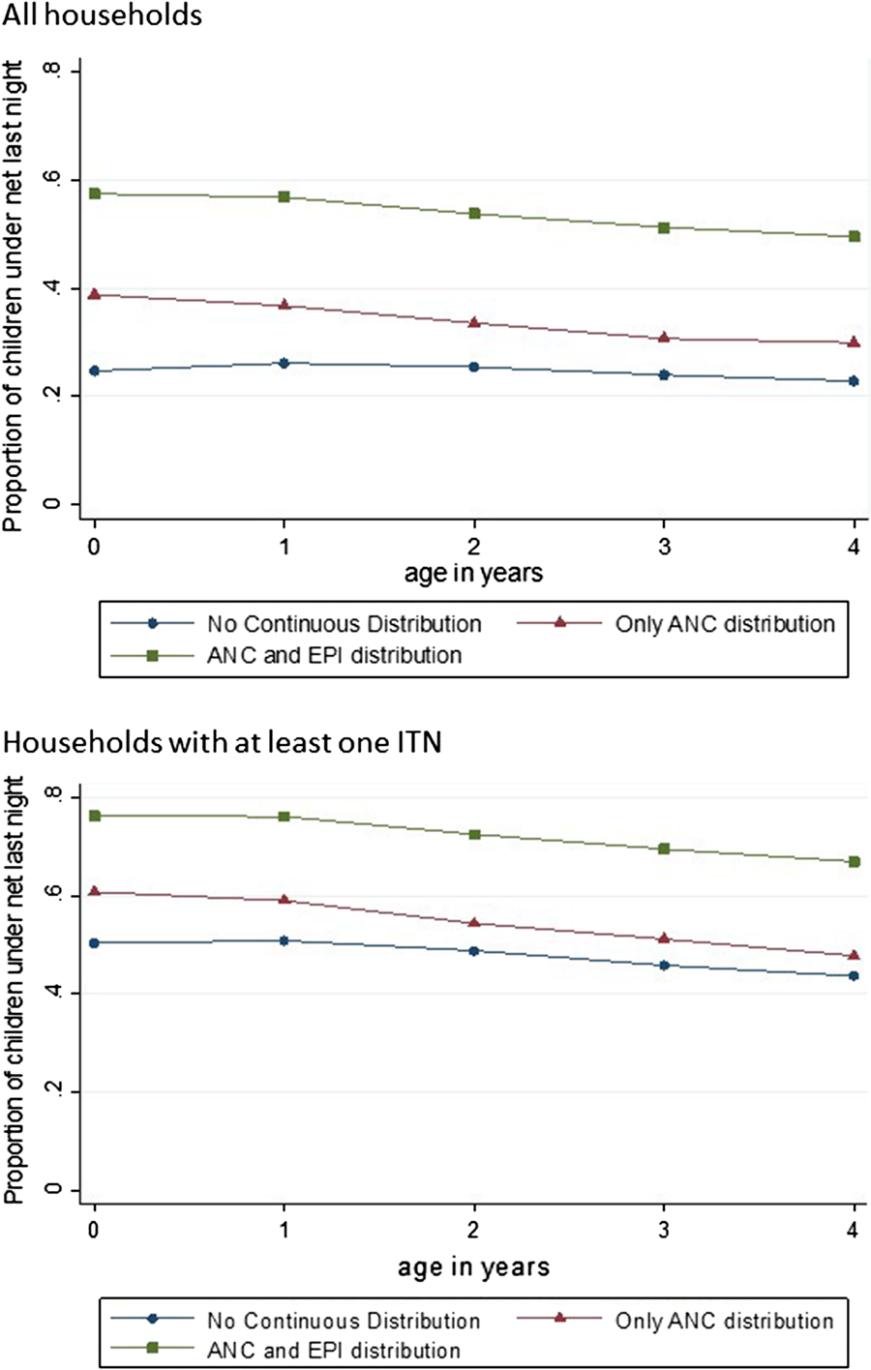
Net use by age, stratified by continuous distribution programme, in all households, and in households with at least one ITN. Countries weighted equally, not by population, to crease estimations for “average country experience” based on the policy options

When net use in children is pooled across countries, (with countries weighted equally to present the average country experience for each policy), the relationship between national policy and children’s net use is seen. Children living in countries with both an ANC and an EPI-based distribution policy were significantly more likely to be sleeping under an ITN, compared to children in countries with either ANC distribution only or no facility-based distribution policy, on average, for all age groups under-five (Fig. 3). The same relationship is seen when looking only at households with at least one ITN (Fig. 3).

Looking closely at the addition of each distribution policy for only children under 1, there appears to be a stepwise improvement in ITN use with the addition of each distribution policy. In children older than 4, by comparison, the addition of the EPI-based distribution policy seems to have a greater effect than the addition of the ANC-based distribution policy (Table 3). Thus infants benefit most from the addition of ITN distributed via ANC, while 4 year olds benefit most from ITNs distributed via both ANC and EPI, compared to only ANC (Table 3).

**Table 3:** Odds ratio (OR) of ITN use by age, in all countries pooled, with ANC-based distribution only as a reference category, for all households and households with at least one ITN, controlling for maternal education and household size

Age (years)	All households			Households with at least 1 ITN		
	No continuous distribution	ANC only	ANC and EPI	No continuous distribution	ANC only	ANC and EPI
< 1	0.52 (0.48–0.57)	Ref	2.12 (1.98–2.27)	0.68 (0.61–0.76)	Ref	2.06 (1.90–2.23)
4	0.71 (0.64–0.78)	Ref	2.28 (2.12–2.45)	0.88 (0.78–0.99)	Ref	2.21 (2.04–2.39)

## Discussion

Net use across countries decreases with age in children under 5 years (Table 1) [15–17]. The decline in net use early in life is a concern for malaria prevention programmes, as these children are more vulnerable to severe malaria than their adult counterparts. The results from this analysis suggest that countries implementing both ANC and EPI based ITN distribution have higher household ITN ownership, higher household universal access, higher ITN use in all children under-five, and higher ITN use in children under-five in houses with at least one ITN, on average, compared with countries with fewer routine facility-based distribution channels. These data suggest that the combined impact of these two facility-based distribution strategies together far surpasses the benefit of ANC-based distribution alone.

Many studies have found ANC to be a useful and/or cost effective ITN delivery strategy [10, 18–26]. A few studies define ANC and EPI based distribution as the same thing, referring to them collectively as “facility-based distribution”, without differentiating between them [11, 27]. This is the first study looking at the separate and cumulative effects of ANC and EPI-based routine distribution compared to the singular effect of just one. One important function of ANC-distributed ITNs is “catch-up and keep-up”: the idea that between campaigns, ANC nets maintain ITN coverage as older nets fall out of use [19, 28]. This may not take into account the fact that in households with pregnancies and births, the household size is increasing, so a “keep-up” strategy might replace used nets, but may not be sufficient to provide additional ITNs to cover the growing household size. Countries providing ITNs via both ANC and EPI are providing additional nets for the increased household size resulting from births as well as ensuring there are enough nets for household members in-between campaigns. This may be why there is such a difference seen between the average country with both policies, compared to the average country with only ANC-based distribution.

Most efforts to increase household ITN ownership have focused on mass-distribution campaigns, while routine facility-based distribution has been seen as an appropriate way to “keep-up” [19]. Mass campaigns are an essential tool for increasing ownership of ITN for vector control, but these findings show that routine facility-based distribution can also be used to increase household ITN ownership. Not only does household ownership, and household universal access, improve with ITN distribution through both ANC and EPI (Table 2), but there is also an increase in ITN use by under-fives (Fig. 1). On a population level, the proportion of total children sleeping under ITNs is higher with the addition of an ITN distribution policy through EPI. More interestingly, even in households with only one net, ITN use among under-five children is higher in countries with than without both ANC and EPI distribution (Fig. 1). This may be a result of health facility training and behaviour change messaging that comes with a policy to distribute ITNs via EPI. Even if ITN availability is not consistent through these channels [8], health workers gain training and implement messaging about net use in infancy, which may be enhanced in countries with EPI-based distribution.

The benefits of ANC-based distribution seem to decrease as children age, but the added benefit from EPI-based distribution increases as children age (Table 3). This may be the result of ANC-distributed ITNs wearing out by the time children reach older ages. If ITNs are expected to last for 3 years, on average [29, 30], a child of 3 or 4 years of age is more likely to have a usable ITN if it was given to them within their first year of life, than if it was given to their mother at some time before they were born.

This analysis is not without limitations. Understanding the consistency and extent of routine facility-based distribution programmes through ANC and EPI is challenging. There are limited data available on national programme implementation, and there may be inconsistencies in reporting to WHO [8]. In countries with reported facility-based distribution, research suggests that ITNs may not be available for the majority of women and children attending these services [8]. While pooled analyses and summary findings have been presented, there is diversity in national ITN coverage and use, within each distribution policy category, especially between countries with no routine facility-based distribution. Countries that have implemented policies for ITN distribution through ANC and/or EPI may differ from countries without these policies.

There is also likely to be significant heterogeneity within one country (regionally, or in urban *vs* rural areas), in terms of how these policies are implemented and the impact of the policies on ITN use. It would be interesting to evaluate one national programme and compare ITN use within households that did and did not receive a net through these channels, but data on net source are not currently available in the DHS. It would also be interesting to follow a country’s ITN use before and after the introduction of an ITN distribution policy, but longitudinal data of that nature are not available.

For countries with only an ANC distribution policy, Nigeria serves as a significant outlier. Net ownership in Nigeria is very similar to that in other countries in this category, but net use in children under-five in Nigeria is appreciably lower. For the pooled estimates, presented in Fig. 3 and Table 3, the low net use in the “Only ANC distribution” group is partially the result of the low ITN use in Nigeria. There were no countries with EPI based distribution, but not ANC based distribution, making it impossible to compare the two programmes individually.

This analysis considered country level patterns in net use based on country self-reporting of policies to the WHO. In order to understand the added benefit of ITNs distributed through ANC vs EPI on an individual or household level, it would be useful to examine ITN ownership within countries, and compare households and families that did and did not receive ITNs through these channels. Unfortunately, the DHS does not currently collect information on ITN distribution via ANC and EPI, for analysis, making that type of analysis impossible.

The logistic analyses controlled for household size and maternal education between countries, but data on broader programmatic or implementation strengths were not available as potential confounders within the DHS dataset. More consistent and robust data on these channels are needed to understand fully the impact they have on ITN ownership and use.

Many countries are still lacking any policy or active distribution of LLINs via ANC and EPI programmes despite WHO recommendations for routine distribution through these channels [8]. These findings suggest that there may be a significant benefit to ITN distribution through both channels, beyond the benefit from ANC distribution alone. Both these distribution channels can be implemented across countries to improve ITN ownership and use.

## Conclusion

As supplements to mass-distribution campaigns, ITN distribution through ANC and EPI, together, can increase net ownership, universal access, and net use in children under-five. These routine distribution programmes, when implemented together greatly improve net ownership and use, and provide nets to vulnerable children who may not otherwise be covered. A second facility-based distributed ITN, via EPI, beyond the one given at ANC, has the potential to increase the total ITNs in a household, increasing the number and proportion of homes with universal access, and improving ITN use in children under five. Beyond “keeping-up” ITN coverage, the combination of these services can improve coverage, which is an important tool for the control and elimination of malaria.

## Data Availability

The datasets generated and/or analysed are available in the Demographic and Health Service (DHS) Programme repository, at: https://dhsprogram.com/data/using-datasets-for-analysis.cfm.
